# The influence of age and winter environment on Rumax Bovibox and Bovibox HM supplement intake behavior of winter grazing beef cattle on mixed-grass rangelands

**DOI:** 10.1093/tas/txaa093

**Published:** 2020-12-22

**Authors:** Samuel A Wyffels, Cory T Parsons, Julia M Dafoe, Darrin L Boss, Tyrell P McClain, Boone H Carter, Timothy DelCurto

**Affiliations:** 1 Northern Agricultural Research Center, Montana State University, Havre, MT; 2 Department of Animal and Range Sciences, Montana State University, Bozeman, MT; 3 PerforMix Nutrition Systems, Nampa, ID

## INTRODUCTION

Chronic cold and wind exposure associated with northern winter grazing environments often expose cattle to conditions below their lower critical temperature, resulting in animals increasing their resting metabolic rate and overall energy expenditure in an effort to maintain homeothermy (Webster, 1971; Christopherson et al., 1979; Keren and Olson, 2006). Cattle typically respond to severe cold conditions by increasing intake to meet thermoregulatory demands (Baile and Forbes, 1974; Ames and Ray, 1983; Arnold, 1985). However, low-quality forage limits intake on winter rangelands at northern latitudes. Supplemental protein is often provided to increase intake of dormant forages to meet the nutritional needs of grazing beef cattle and maintain a desired level of productivity (Lusby et al., 1967; Bowman et al., 1995; Bodine et al., 2001). Winter protein supplementation strategies assume that all animals consume a daily target quantity of supplement and deviation from the targeted intake can have negative impacts on animal performance (Bowman and Sowell, 1997). Cow age has been shown to be an influential factor affecting individual supplement intake and foraging behavior (Adams et al., 1986; Kincheloe et al., 2004; Wyffels et al., 2018). However, data are limited relative to sources of variation with beef cattle supplemented in herd groups in extensive winter environments. The lack of information is related to the difficulty in measuring intake of free-choice supplements in extensive production environments (DelCurto and Olson, 2010). Therefore, research is needed to refine supplementation strategies that optimize nutrient delivery to diverse groups of animals in extensive environments.

Potential changes in energetic requirements to maintain homeothermy could alter supplement intake during winter months. Short-term behavioral responses may be critical to the energy balance of domestic animals under extreme weather conditions (Senft and Rittenhouse, 1985). Therefore, the goal of this research is to examine the effects of cow age and winter weather conditions on supplement intake behavior of cattle grazing winter rangelands offered Rumax Bovibox supplement. We hypothesize that supplement intake behavior is altered by the interaction of cow age and winter environmental conditions.

## MATERIALS AND METHODS

The use of animals in this study was approved by the Institutional Animal Care and Use Committee of Montana State University (#2018-AA12).

A commercial herd of nonlactating bred cows (Angus, Simmental × Angus) ranging in age from 1 to 12 yr of age were assigned to one of six age classifications (1-, 2-, 3-, 4-, 5- to 7-, and ≥8-yr-old cattle) and winter grazed on a 329-ha rangeland pasture (~1.5 AUM ha^−1^) for 2 yr (291 cows year 1 and 316 cows year 2) with an average weight of 562.93 kg and body condition of 5.5. The winter grazing period occurred from mid-November to early-January 2018 to 2019 and 2019 to 2020. All cattle had free-choice access to Rumax Bovibox HM (2018 to 2019) and Rumax Bovibox protein blocks (2019 to 2020; Table 1). The daily target supplement intake range was 0.45 to 0.91 kg∙cow^−1^∙d^−1^. Each individual animal was equipped with an electronic ID tag (Allflex USA, Inc., Dallas-Ft. Worth, TX) attached to the interior of the left ear for the measurement of daily individual supplement intake (kg∙cow^−1^∙d^−1^) and time spent at the supplement feeder (min∙d^−1^) using a SmartFeed Pro self-feeder system (C-Lock Inc., Rapid City, SD) with a total of eight feeding stations. Variation in supplement intake, measured as coefficient of variation, was based on daily intake estimates for individual animals. Supplement intake behavior was recorded for 45 d, and individual cow was considered the experimental unit.

**Table 1.** Supplement composition for cattle winter grazing rangeland in 2018 to 2019 and 2019 to 2020 at the Thackeray Ranch, Havre, MT (as-fed basis)

	Bovibox HM^1^	Bovibox^2^
Crude protein	28.7% min	30% min
Crude fat	1.45% min	1.5% min
Crude fiber	5.0% max	5.0% max
Calcium	1.3% min	1.3% min
	1.8% max	1.8% max
Phosphorus	0.7% min	0.7% min
Salt	23% min	23% min
	26% max	26% max
Potassium	1.5% min	1.5% min
Magnesium	2.5% min	1.0% min
Manganese	856 ppm min	880 ppm min
Zinc	1,074 ppm min	1,100 ppm min
Copper	213 ppm min	220 ppm min
Copper (from chelate)	108 ppm min	110 ppm min
Cobalt	15 ppm min	16 ppm min
Iodine	26 ppm min	25 ppm min
Selenium	3.3 ppm min	3.3 ppm min
	3.6 ppm max	3.6 ppm max
Selenium yeast	—	1.7 ppm
Vitamin A	12,000 IU/lb	40,800 IU/lb
Vitamin D	4,000 IU/lb	4,500 IU/lb
Vitamin E	25 IU/lb	50 IU/lb
NPN not more than	9.7%	9.9%

^1^Supplement used 2018 to 019.

^2^Supplement used 2019 to 2020.

An Onset HOBO U30-NRC Weather Station (Bourne, MA) was placed near the supplement feeders and programmed to collect ambient air temperature and wind speed every 15 min for the entirety of the grazing period. Temperatures adjusted for windchill (*T*_windchill_) were calculated using a modified version of the National Weather Service formula (Osczevski and Bluestein, 2005; Tucker et al., 2007; Graunke et al., 2011). Daily average weather conditions were paired with daily supplement intake readings for each individual animal for the duration of the grazing period. Each day was then classified as below average (< −1 SD below the mean), average (±1 SD from mean), or above average (>+1 SD above the mean) *T*_windchill_ within each year of the grazing trial (Table 2).

**Table 2.** Average temperature (°C), wind speed (km∙h^−1^), and temperature adjusted for windchill (*T*_windchill_; °C) below average, average, above average weather conditions, and overall year means (± SE) for the 2 yr of grazing (2018 to 2019, 2019 to 2020) at the Northern Agricultural Research Center Thackeray Ranch, Havre, MT

	Weather classification			Overall mean
	Below average	Average	Above average	
Year 1				
Temperature, °C	−8.10 ± 1.13	0.91 ± 0.61	8.20 ± 0.30	0.58 ± 0.83
Wind speed, km∙h^−1^	5.89 ± 0.70	22.77 ± 0.99	42.56 ± 2.10	22.90 ± 1.64
* T*_windchill_, *°*C	−18.69 ± 1.23	−7.10 ± 0.61	1.46 ± 0.43	−7.17 ± 0.90
Year 2				
Temperature, °C	−9.87 ± 0.54	−0.86 ± 0.57	6.94 ± 1.14	−1.22 ± 0.81
Wind Speed, km∙h^−1^	6.08 ± 0.71	22.20 ± 1.66	42.27 ± 1.20	22.19 ± 1.99
* T*_windchill,_ °C	−17.89 ± 0.74	−8.31 ± 0.53	−0.15 ± 1.33	−9.12 ± 0.84

This study was conducted at the Thackeray Ranch (48°21′N 109°30′W), part of the Montana Agricultural Experiment Station located 21 km south of Havre, MT. Climate is characterized as semi-arid steppe with an average annual precipitation of 410 mm. Vegetation is dominated by Kentucky bluegrass (*Poa pratensis* L.), bluebunch wheatgrass (*Pseudoregnaria spicata* [Pursh] A. Love), and rough fescue (*Festuca scabrella* Torr.). The production and quality of pasture vegetation was estimated by sampling 10 randomly located plots prior to grazing each year. Clipped samples were placed in a forced air oven at 60 °C for 48 h and then weighed. Vegetation samples from each plot were ground to pass a 1-mm screen in a Wiley mill and sent to a commercial lab to be analyzed for crude protein, neutral detergent fiber, acid detergent fiber, and total digestible nutrients as indicators of vegetation quality (Dairy One, Ithaca, NY; Table 3).

**Table 3.** Average annual grass production (kg∙ha^−1^), crude protein (CP, %), neutral detergent fiber (NDF; %), acid detergent fiber (ADF; %), and total digestible nutrients (TDN; %) of the experimental paddock for the 2 yr of grazing (2018 to 2019, 2019 to 2020) at the Northern Agricultural Research Center Thackeray Ranch, Havre, MT

	Production (kg∙ha^−1^)	CP (%)	NDF (%)	ADF (%)	TDN (%)
Year 1	1,790	5.4	63.2	41.9	56.0
Year 2	1,456	5.4	66.9	39.9	55.0

Daily individual supplement intake, the coefficient of variation (CV) of supplement intake, and time spent at the supplement feeder were analyzed using ANOVA with a generalized linear mixed model including cow age, year, *T*_windchill_, *T*_windchill_ × cow age, cow age × year, *T*_windchill_ × year, and *T*_windchill_ × cow age × year as fixed effects, and individual cow as the random effect. An alpha ≤ 0.05 was considered significant. Orthogonal polynomial contrasts were used to determine linear and quadratic effects for each analysis. Means were separated using the Tukey method when *P* < 0.05. All statistical analyses were performed in R (R Core Team, 2017).

## RESULTS

**Figure 1.**
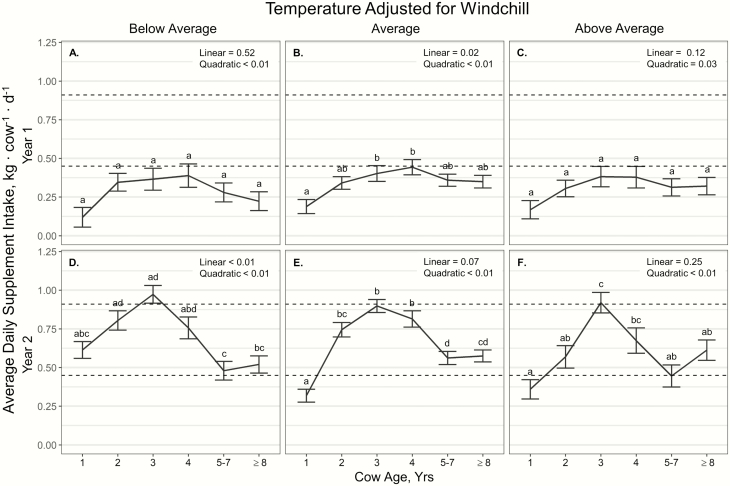
Influence of cow age, year, and temperature classification (adjusted for windchill) on average daily supplement intake (kg∙cow^−1^∙d^−1^; ± SE; target intake range indicated by dashed lines) by cattle grazing dormant northern mixed grass rangeland in 2018 to 2019 and 2019 to 2020 at the Northern Agricultural Research Center Thackeray Ranch, Havre, MT.

Average daily supplement intake displayed a *T*_windchill_ × cow age × year interaction (*P* = 0.02; Figure 1). In year 1, there was no effect of age on daily supplement intake at below average and above average *T*_windchill_ (*P* ≥ 0.07; Figure 1A and C). Age displayed a quadratic effect on daily supplement intake at average *T*_windchill_ in year 1 (*P* < 0.01; Figure 1B); however, this effect was limited to 3- and 4-yr-old cattle consuming more supplement per day than yearlings (*P* ≤ 0.02). In year 2, cow age had quadratic effects on supplement intake for all *T*_windchill_ conditions (*P* < 0.01; Figure 1D–F). At below average *T*_windchill_ conditions in year 2, 2- and 3-yr-old cattle consumed more supplement per day than 5- to 7- and ≥8-yr-old cattle (*P* < 0.01; Figure 1D). Average *T*_windchill_ conditions in year 2 resulted in yearlings consuming less supplement than all other age groups (*P* < 0.01), whereas 3- and 4-yr-old cattle consumed more supplement per day than 5- to 7- and ≥8-yr-old cattle (*P* < 0.01; Figure 1E). At above average *T*_windchill_ conditions in year 2, 3-yr-old cattle consumed more supplement per day than yearlings, 2-, 5- to 7-, and ≥ 8-yr-old cattle (*P* ≤ 0.01), with 4-yr-old cattle consuming more supplement per day than yearlings (*P* = 0.03; Figure 1F).

**Figure 2.**
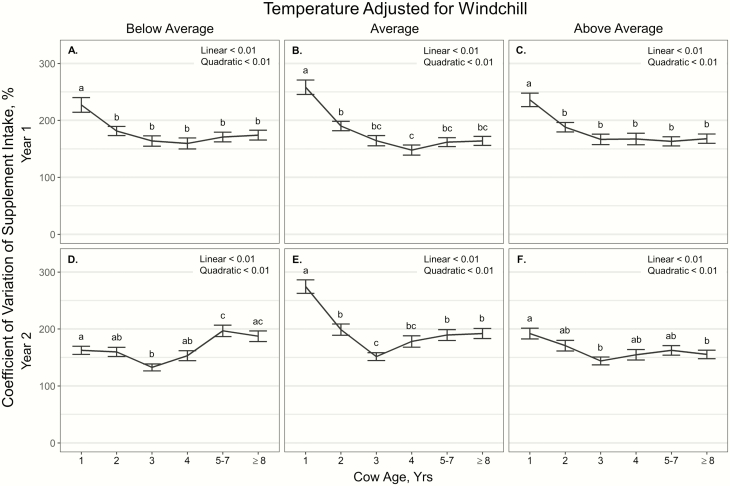
Influence of cow age, year, and temperature classification (adjusted for windchill) on the coefficient of variation of supplement intake (%; ± SE) by cattle grazing dormant northern mixed grass rangeland in 2018 to 2019 and 2019 to 2020 at the Northern Agricultural Research Center Thackeray Ranch, Havre, MT.

Variation in supplement intake (% CV) also displayed a *T*_windchill_ × cow age × year interaction (*P* = 0.05; Figure 2), where age exhibited quadratic effects on variation in supplement intake across all *T*_windchill_ conditions during both years (*P* < 0.01). However, the quadratic effects of age in year 1 were limited to yearlings having higher variation in supplement intake than all other age classes of cattle (*P* < 0.01; Figure 2A–C), with 4-yr-old cattle having less variation in supplement intake than 2-yr-old cattle during average *T*_windchill_ conditions (*P* = 0.01; Figure 2B). In year 2, at below *T*_windchill_ conditions, 3-yr-old cattle had less variation in supplement intake than yearlings, 5- to 7-, and ≥8-yr-old cattle (*P* ≤ 0.02) with yearlings having less variation than 5- to 7-yr-old cattle (*P* = 0.05; Figure 2D). At average *T*_windchill_ conditions in year 2, yearlings had higher variation in supplement intake than all other age categories (*P* < 0.01), with 3-yr-old cattle having less variation in supplement intake than 2-, 5- to 7-, and ≥8-yr-old cattle (*P* ≤ 0.01; Figure 2E). At above average *T*_windchill_ conditions in year 2, 3- and ≥8-yr-old cattle had less variation in supplement intake than yearlings (*P* = 0.03; Figure 2F).

**Figure 3.**
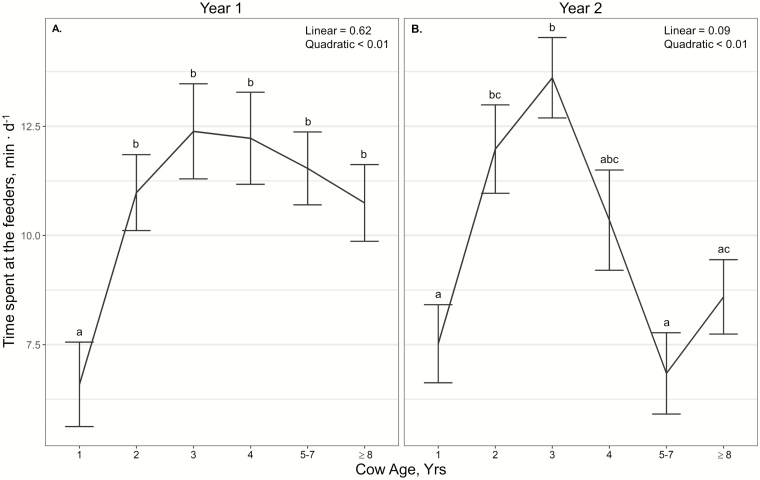
Influence of cow age and year on average time spent at the supplement feeder (min∙d^−1^; ± SE) by cattle grazing dormant northern mixed grass rangeland in 2018 to 2019 and 2019 to 2020 at the Northern Agricultural Research Center Thackeray Ranch, Havre, MT.

Time spent at the feeder per day exhibited a year × cow age interaction (*P* < 0.01; Figure 3A and B). Cow age displayed quadratic effects on time spent at the feeder per day in both years (*P* < 0.01); however, effects in year 1 were limited to yearlings spending less time at the feeder than all other cattle (*P* ≤ 0.01; Figure 3A). In year 2, 3-yr-old cattle spent more time at the supplement feeders than yearlings, 5- to 7-, and ≥8-yr-old cattle, with 2-yr-old cattle spending more time at the feeders than yearlings and 5- to 7-yr-old cattle (*P* ≤ 0.01; Figure 3B). Time spent at the feeder per day also displayed a year × *T*_windchill_ interaction (*P* < 0.01). In year 1, cattle spent more time at the feeder during average *T*_windchill_ conditions (12.24 ± 0.42 min) than below and above average (*P* < 0.01; 9.43 ± 0.42 and 10.57 ± 0.54 min), with no effect of *T*_windchill_ on time spent at the feeder in year 2 (*P* ≥ 0.08).

## DISCUSSION

The results of our study suggest that the interaction of cow age, winter weather conditions, and year can have a significant impact on supplement intake behavior. However, the year effects observed for all supplement intake variables is likely related to the difference between Rumax Bovibox HM and Bovibox as weather and forage conditions were similar for both years of the study. Both Rumax Bovibox HM and Bovibox supplements are formulated similarly, however, Rumax Bovibox HM contains 2.5% MgO (1.0% in Bovibox), which increases bitterness/hardness and may have led to cattle consuming below the target supplement intake range the first year of our study. Furthermore, average supplement intake in year 1 was approximately 50% than that of year 2, whereas time at the feeders were similar for both years.

Past research that quantified supplement intake behavior of mixed age herds of cattle have found that older cows typically consume more supplement and are less variable in their daily supplement intake than younger cows (Bowman et al., 1999; Sowell et al., 2003; Kincheloe et al., 2004). Our results contradict this conventional idea as we found 3- to 4-yr-old cattle to have the highest supplement intake with the least variation across weather conditions. Recent research evaluating the effects of winter weather conditions and cow age on supplement intake behavior suggests that supplement intake decreases with cow age at cold temperatures (Wyffels et al., 2018). Although the effects observed in our study were quadratic in nature, yearling cattle did increase Rumax Bovibox intake with decreased variation during below average temperature conditions. However, yearling cattle only consumed target supplement intake with Rumax Bovibox at below average temperature conditions and were highly variable consumers suggesting that supplementing with self-fed protein blocks in a mixed-aged cow herd may not provide adequate nutrients for yearlings.
